# Dr. Fenner’s Southern Reports

**Published:** 1851-10

**Authors:** 


### DR. FENNER'S SOUTHERN REPORTS.

We regret that a notice of this valuable work has been crowded out of this number of the Journal. Dr. Fenner deserves great praise and encouragement for the contributions he is making to medical science, by the publication of his valuable annual. He should receive the aid of all who feel any desire to sustain and cherish valuable medical enterprises. Dr. Fenner's work is published at two dollars and fifty cents, and it is richly worth its cost to any one who feels interested in the advancement of the medical profession.

In a future number, we shall endeavor to make our readers better acquainted with the character of Dr. Fenner's Southern Medical Reports.
B.

